# A rare presentation of blastomycosis as a multi-focal infection involving the spine, pleura, lungs, and psoas muscles in a Saudi male patient: a case report

**DOI:** 10.1186/s12879-022-07203-x

**Published:** 2022-03-07

**Authors:** Safwat Eldaabossi, Mustafa Saad, Hameed Aljawad, Badr Almuhainy

**Affiliations:** 1 Division of Pulmonary, Almoosa Specialist Hospital, Al Ahsa, Kingdom of Saudi Arabia; 2 Department of Chest Diseases, Alazhar University, Cairo, Egypt; 3 Division of Infectious Diseases, Department of Internal Medicine, Almoosa Specialist Hospital, Al Ahsa, Kingdom of Saudi Arabia; 4 Division of Pathology, Department of Laboratory Medicine, Almoosa Specialist Hospital, Al Ahsa, Kingdom of Saudi Arabia; 5 Division of Infectious Diseases, Department of Internal Medicine, King Fahad Hospital, Al Ahsa, Kingdom of Saudi Arabia

**Keywords:** *Blastomycosis*, Pleural effusion, Pulmonary nodules, Psoas muscles

## Abstract

**Background:**

Blastomycosis is a disease caused by the fungus *Blastomyces*—a thermally dimorphic fungus that can cause granulomatous and/or purulent infection.

**Case presentation:**

We report here a case of chronic *blastomycosis* infection in a 24-year-old male patient from Saudi Arabia who presented with recurrent skin abscesses associated with deep-seated and multilevel paraspinal (dorsal and lumbar) collections and bilateral empyema with pulmonary involvement and bilateral psoas abscesses. The diagnosis was made after a CT-guided pleural biopsy revealed the characteristic histopathological findings of blastomycosis. The patient underwent several drainage procedures and was successfully treated with a long-term course of oral itraconazole.

**Conclusions:**

Chronic *blastomycosis* may have clinical and radiologic features similar to thoracic tuberculosis or malignant disease. There is no definite clinical symptom of blastomycosis, and thus a high degree of suspicion is required for early diagnosis. This case is a rare form of *blastomycosis* with chronic multifocal purulent infection and is the second case of *blastomycosis* reported in Saudi Arabia.

## Background

Empyema thoracica is an infection of the pleural space. Empyema is usually due to an underlying infection of the lung parenchyma, but may also be caused by a blood-borne infection, thoracic surgery, trauma, abdominal infection, or neoplasm. Empyema is common and a leading cause of morbidity and mortality worldwide. The most common cause of empyema is a bacterium (anaerobic and aerobic). *Tubercle bacilli*, *Nocardia asteroides*, and fungi are increasingly also detected. blastomycosis rarely causes empyema and is caused by the fungus *Blastomyces dermatitidis*. It can present as chronic granulomatous and purulent mycosis. It is often a dimorphic fungus that occurs naturally as a nonpathogenic mold in the mycelial form and converts to a pathogenic yeast at body temperature. Infection is acquired either via inhalation or inoculation. We report a case of *blastomycosis* with bilateral empyema, bilateral psoas collection, and pulmonary findings in a 24-year-old Saudi male. He presented with a dry cough, shortness of breath, and bilateral pleural effusion that did not resolve with antibiotic and anti-tuberculous treatment. The diagnosis was made via a CT-guided pleural biopsy that showed yeast as per North American blastomycosis. After treatment with itraconazole, the patient was symptom-free and resumed his previous activities. This is the second published case of blastomycosis in Saudi Arabia.

## Case presentation

**Fig. 1:**
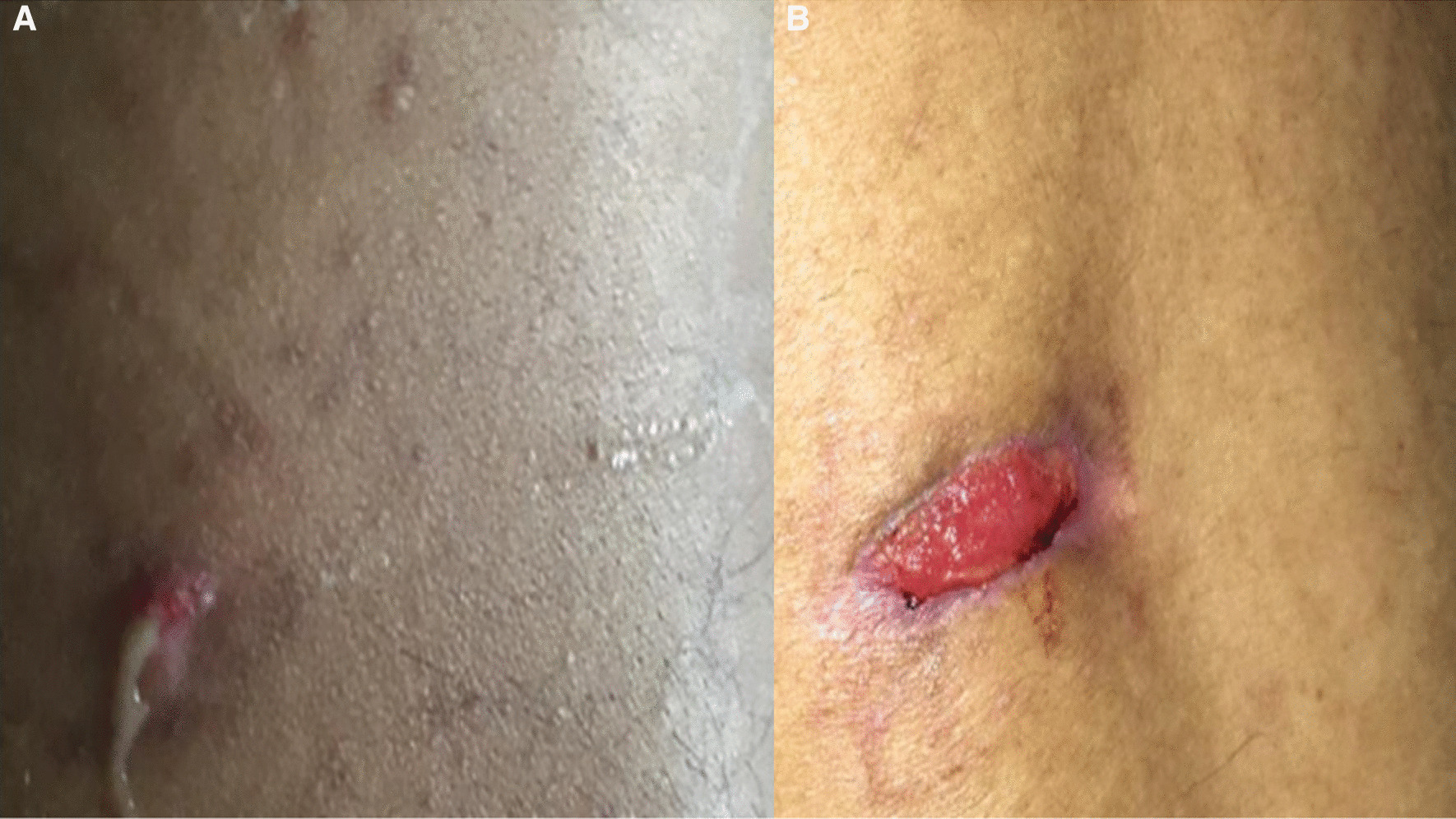
**a**, **b** The recurrent cutaneous abscesses

We report a 24-year-old Saudi male patient who was previously healthy. His history dates back to April 2018 when he developed a superficial abscess in the inferior para-dorsal region (Fig. 1a, b) with dry cough, fatigue, and profuse sweating but no fever or chills. He was living in the southern part of the state of Kentucky in the USA where he studied for about three years. He was examined at a local surgical clinic and received incision and drainage of the abscess followed by oral antibiotic therapy. After partial improvement, the abscess recurred within three months, and he underwent a second incision and drainage followed by another course of oral antibiotics that brought only partial improvement. He returned to Saudi Arabia a few months later. Shortly after his arrival, abscess formation recurred for the third time in the same region. He was treated at an internal medicine clinic and received prolonged treatment with oral antibiotics plus I&D for the third time. The abscess did not subsequently recur, but his respiratory symptoms and excessive sweating worsened with additional exertional dyspnea and a weight loss of approximately 8 kg. He was admitted to another hospital in our region in October 2019 where he had some worrisome findings on imaging; he was referred then to our hospital for further diagnostic workup.

**Fig. 2:**
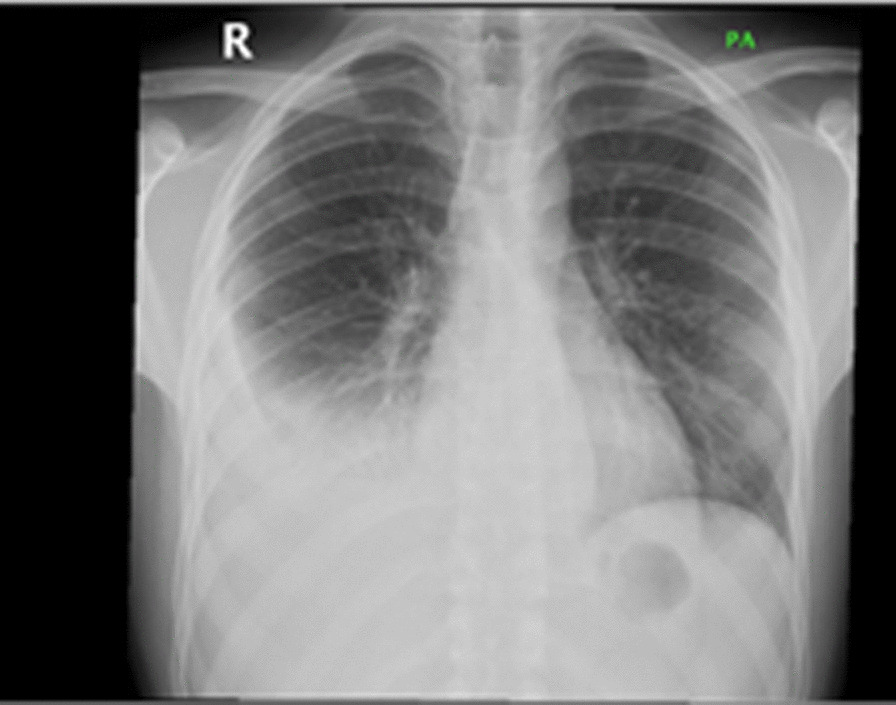
Chest X-ray showing right pleural effusion

**Fig. 3:**
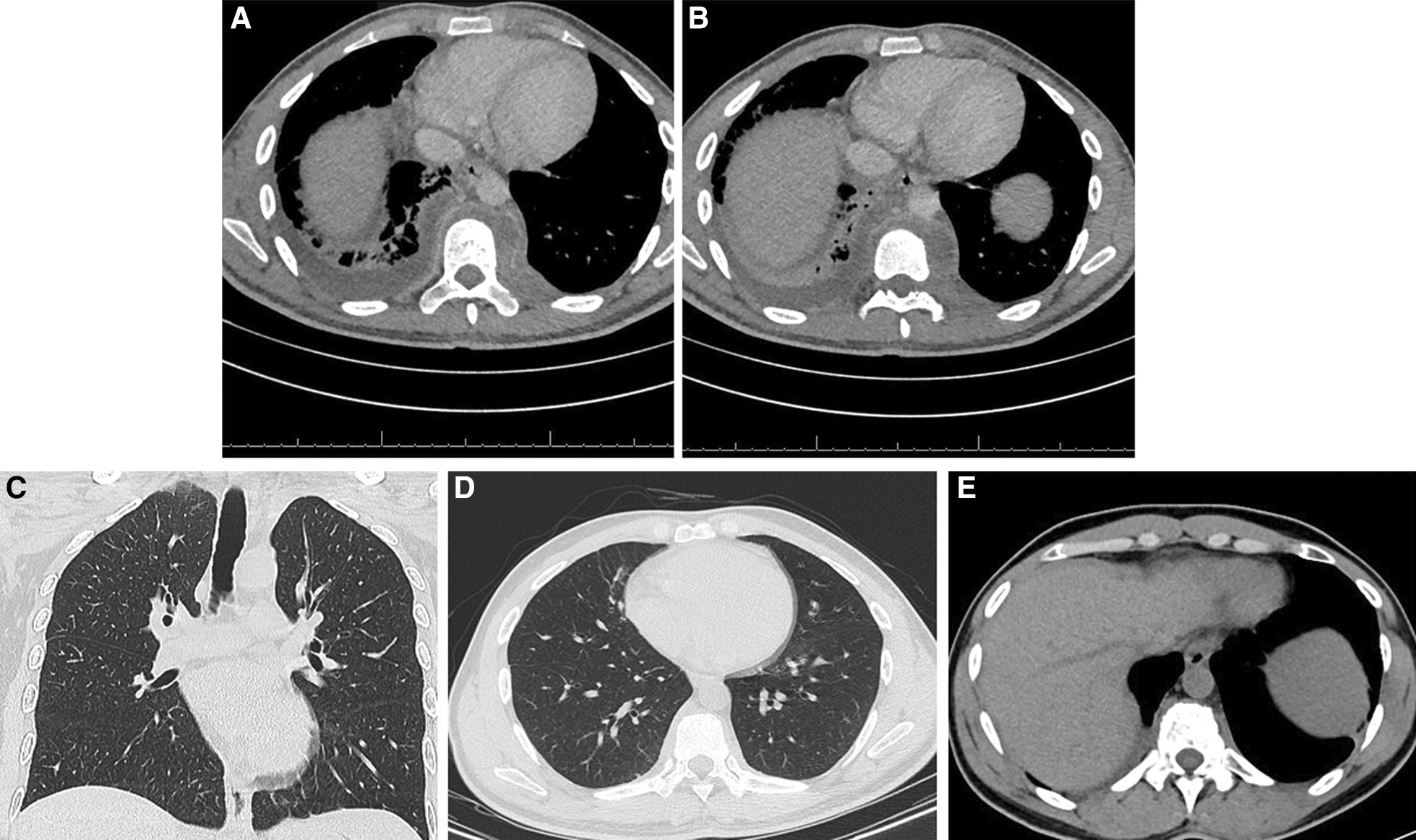
**a**, **b** Chest CT scan showing bilateral effusions more over the right, right segmental infiltrates, and loculated paraspinal collections. **c**, **d**, and **e** Follow up CT scans of the chest showing complete resolution of the empyema and paraspinal collections

**Fig. 4:**
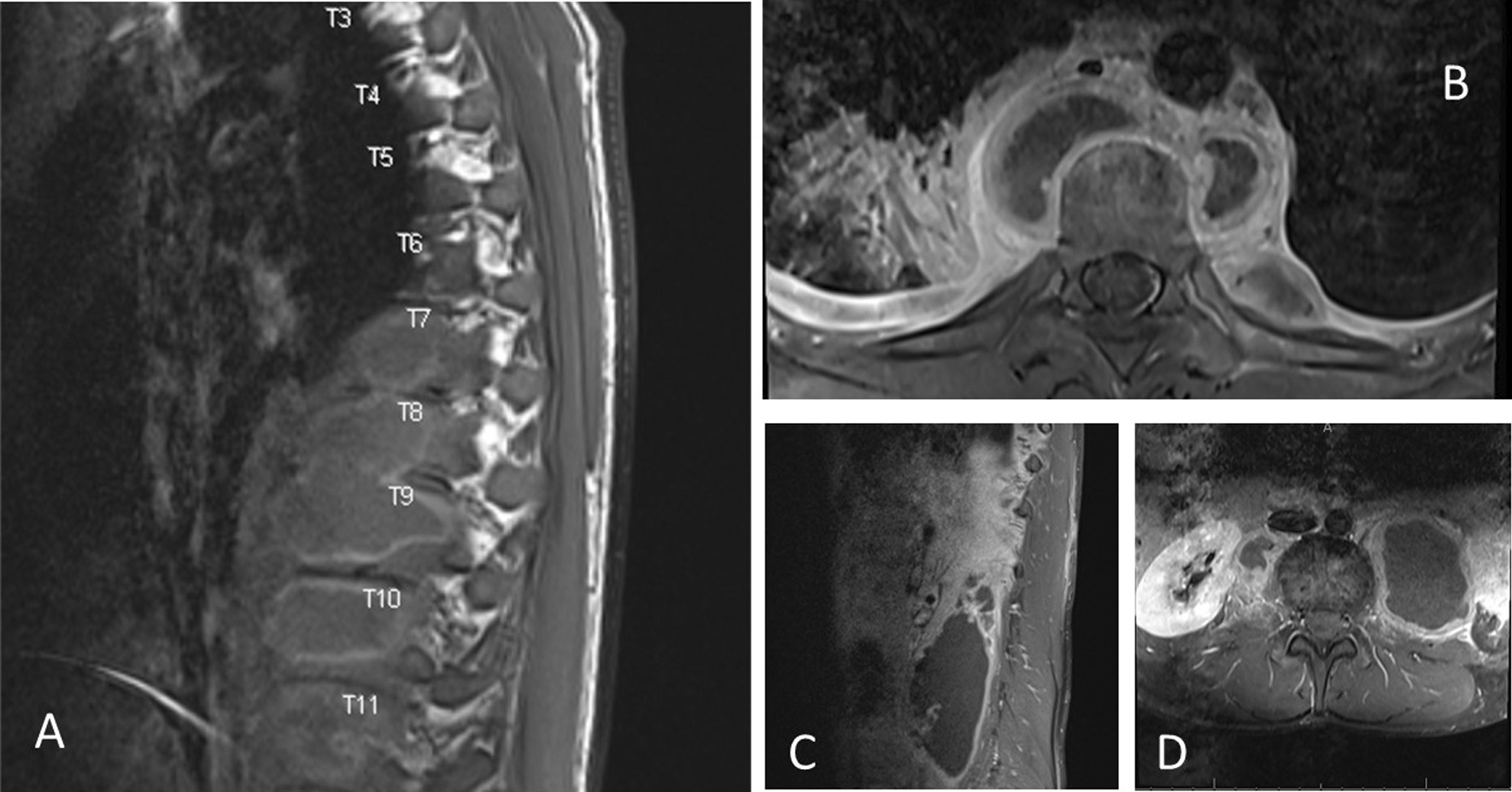
**a**, **b** MRI of the spine showing multi-level paraspinal collections, and psoas abscesses (**c** and **d**)

He presented as thin but not in distress with normal oxygen saturation on room air. His vital signs were stable. Auscultation of the chest showed decreased air entry at both lung bases especially on the right side. There were also multiple scars from previous I&D in the left para-dorsal area. The rest of the physical examination was unremarkable. Laboratory tests revealed a leukocyte count (WBC) of 8.2 × 109/L, hemoglobin of 10.8 g/L, MCV of 73.2 fL, platelet count of 375 × 109/L, C-reactive protein of 248 mg/L, erythrocyte sedimentation rate of 92 mm/H, and alanine aminotransferase of 53 U/L. Serum electrolytes, renal function, albumin levels, and aspartate aminotransferase were all normal. Serology for *Brucella* and *HIV* was negative, and a skin test with purified protein derivatives (PPD) was also negative. A chest radiograph showed pleural effusion on the right side (Fig. 2) while a chest CT showed bilateral effusions higher on the right side, segmental infiltrates in the right lower lobe, and multiple localized paraspinal collections (Fig. 3a, b). An MRI of the dorsal and lumbar spine showed abnormal medullary signal intensity and enhancement in multiple vertebral bodies and posterior elements that included the T8 to L1 vertebrae with multiloculated paravertebral abscesses predominantly on the left side. There was a large left (and a smaller right) psoas muscle abscess (Fig. 4a–d). MRI showed no intradural extension or compression of the spinal cord. The patient underwent right ultrasound-guided thoracentesis with a pigtail catheter through which approximately 900 mL of purulent fluid was drained. Pleural fluid analysis indicated pH 7.0, glucose 60.1 mmol/L, protein 105 g/L, lactate dehydrogenase 1456 U/L, and WBC 26,000 cells/µL of which 84% were neutrophils. Cytology showed no malignant cells. Gram staining of the pleural fluid showed no bacteria; bacterial and fungal cultures were negative, and a smear of pleural fluid with acid-fast bacilli and a polymerase chain reaction (PCR) test for tuberculosis (TB) were negative. A tuberculosis culture (TB) was not performed.

**Fig. 5:**
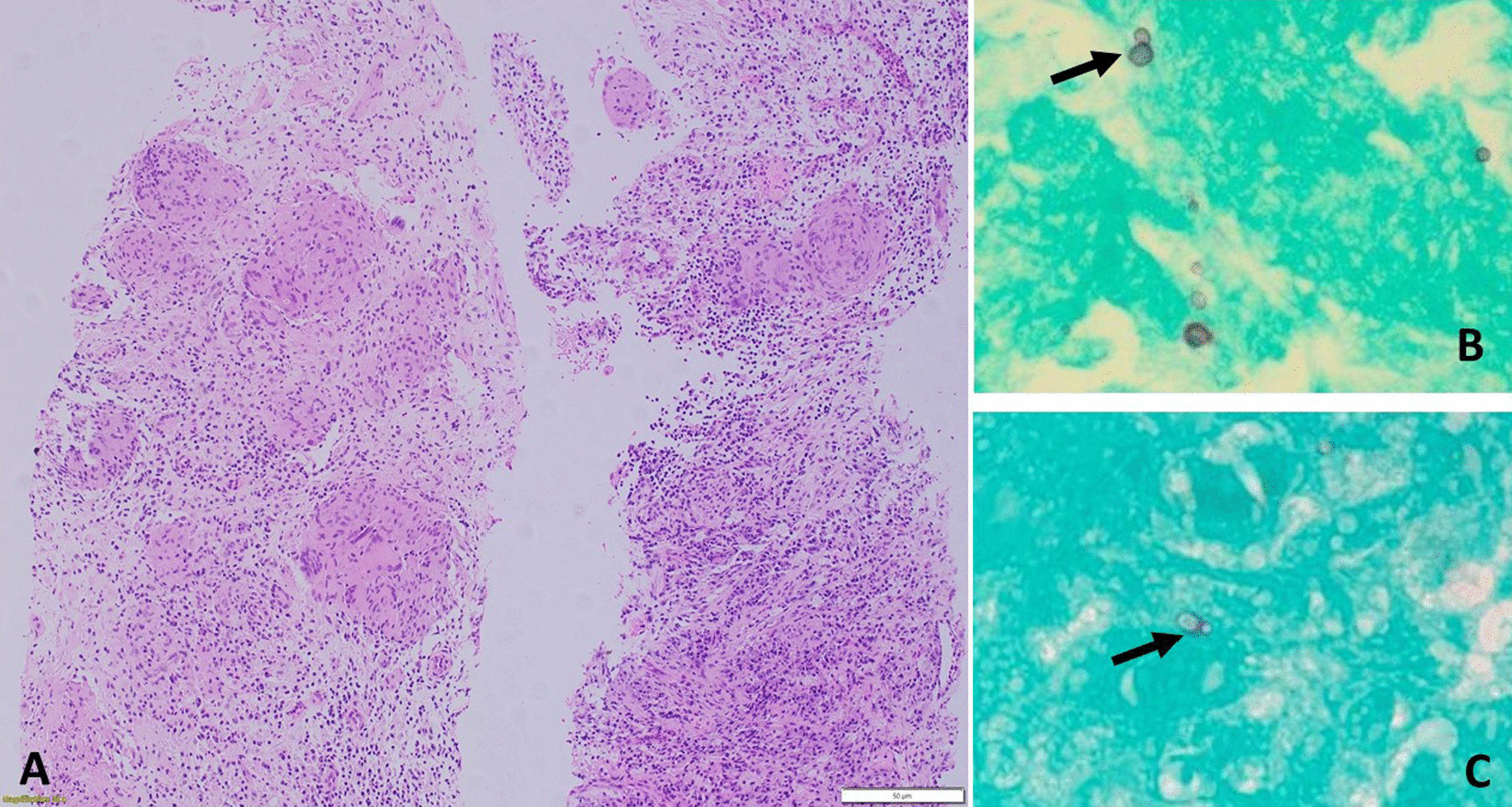
**a**, **b**, and **c** H&E stain and low-power view shows granulomatous lesions characterized by aggregates of epithelioid histiocytes, multinucleated giant cells, and inflammatory background predominated by lymphocytes and plasma cells with sprinkling of neutrophils. No necrosis or fibrin deposition was seen. **B** and **c** GMS stain, high power views show yeast forms with broad based budding (black arrows)

Given the clinical findings without a confirmed diagnosis, the patient was offered a trial of four-drug therapy for TB (rifampicin 600 mg, isoniazid 300 mg, ethambutol 800 mg, and pyrazinamide 1 g orally once daily, and pyridoxine 100 mg daily) until the TB cultures were completed. He was discharged to his referring hospital. He was readmitted to the referring hospital within a month for treatment of liver toxicities associated with the anti-TB drugs. Anti-TB therapy was continued for approximately two more months and discontinued after final TB culture results showed no growth. The patient's waxing and waning symptoms persisted and due to the re-accumulation of pleural fluid and left psoas abscess. A second drainage was performed, but cultures and cytology were again negative. The patient was treated with linezolid (600 mg i.v.) and ceftriaxone (2 g daily i.v. for 2 weeks) followed by linezolid (600 mg p.o. twice daily for three weeks). This led to only partial improvement. In April 2020, the patient was referred to our hospital for the second time because of recurrent pleural effusions and persistence of para-vertebral collections and left psoas abscess. Here, he underwent another CT-guided drainage but this time with a pleural core biopsy. All culture results from the fluid and tissue showed no growth, but the pleural fluid was positive for yeast and tissue histopathology showed granulomatous inflammation and multiple, broad-based, budding yeast structures consistent with blastomycosis (Fig. 5a–c). A diagnosis of multifocal *blastomycosis* was made, and the patient was offered treatment with liposomal amphotericin B in view of the multifocal and extensive bone and tissue involvement in the chest and abdomen; however, he refused to prolong his hospital stay and declined this treatment.

His symptoms began to improve 2 to 3 weeks after starting treatment with itraconazole 200 mg orally twice daily with marked improvement in his respiratory symptoms and cessation of excessive sweating; he began to gain weight. The patient tolerated itraconazole well, and close monitoring of liver enzymes revealed no increase over time. The patient did not require any additional drainage procedures over the next few months. Approximately four months after initiation of therapy, the patient underwent a repeat CT examination of the chest, abdomen, and pelvis, which showed complete resolution of pleural effusions, pulmonary infiltrates, and psoas abscesses (Fig. 3c, d, e). The patient received itraconazole (200 mg orally twice daily) for 12 months due to vertebral involvement; he then completed itraconazole therapy on April 23, 2021 with complete clinical and radiological recovery.

## Discussion and conclusions

Given the clinical findings without a confirmed diagnosis, the patient was offered a trial of 4-drug therapy for TB until the results of the TB cultures were available. He was discharged to his referring hospital shortly thereafter. However, he was readmitted to the referring hospital within a month for treatment of liver toxicities associated with the anti-TB drugs. Anti- TB therapy was continued for approximately two months and discontinued after the final TB culture results showed no growth.

Extrapulmonary tuberculosis (tuberculous pleurisy, tuberculous meningitis, tuberculous lymphadenitis, urogenital tuberculosis, and Pott's disease of the spine) occurs in 15–20% of active cases when the infection spreads outside the lungs [1]. The incidence rate of tuberculosis in Saudi Arabia in 2019 was 9.9/100,000; the country has a moderate burden of TB infection. The infection rate of extrapulmonary tuberculosis in Saudi Arabia remains higher than the global rate of 30% for other developing countries.

Saudi Arabia is facing difficulties in the clinical management of extrapulmonary tuberculosis because differential diagnosis is challenging. Also the integration of modern diagnostic technologies in all centers is still ongoing, and there are difficulties in obtaining an adequate sample [2]. The gold standard for the diagnosis of extrapulmonary tuberculosis remains the detection of *Mycobacterium tuberculosis* in fluid or biopsy specimens either by microscopy and/or culture. Histology includes the study of caseating granulomas together with acid-fast bacilli [1]. Empiric anti-tuberculous treatment may be started with isoniazid, rifampin, and pyrazinamide for two months followed by four months of two drugs (isoniazid and rifampin) until the final diagnosis of extrapulmonary tuberculosis can be confirmed [1, 2].

The clinical presentation of *blastomycosis* can range from asymptomatic infection in the immunocompetent host to disseminated disease in the immunocompromised patient. In addition, the clinical presentation of blastomycosis may mimic other diseases, thus resulting in delayed diagnosis and treatment—especially when patients present in countries outside regions where blastomycosis is known to be endemic. In the United States, *blastomycosis* usually presents as pulmonary disease occurring in 82–93% of patients with cutaneous and osteoarticular involvement in 18–21% and 4–15% of patients, respectively [3]. Outside the US, the clinical picture may be quite different. In a review of 143 patients from Africa, blastomycosis was found to most commonly affect the skin (73.0%), followed by the lungs (56.7%) and bones/joints (46.9%). Multifocal infections and pleural effusions are rare presentations of *blastomycosis* [4]. Our patient presented with multi-level involvement of the dorsal and lumbar spine, bilateral pleural effusions, pulmonary involvement, and psoas abscesses.

*Blastomycosis* is a common endemic mycosis. The latter is a group of mycoses that infect people living in certain regions of the world. Most patients acquire blastomycosis from environmental exposure via inhalation or less commonly by direct inoculation. Our patient’s exposure was likely environmental (inhalation of *blastomycosis* spores), which then caused pleuropulmonary infection that spread locally to involve other adjacent areas.

Cytological examination of pleural fluid may aid in the diagnosis of pleural effusion secondary to blastomycosis [5]. In our case, pleural fluid analysis, cytology, and microbiological studies did not bring the diagnosis to attention. The diagnosis was delayed because *blastomycosis* can mimic many other disease processes including bacterial infection, tuberculosis, and malignancy. A high index of suspicion among patients who live in or travel to endemic areas should be maintained in such patients. Once suspected, the diagnosis of blastomycosis can usually be confirmed by demonstration of the characteristic broad-based budding organisms in sputum or tissues in the presence of granulomatous inflammation utilizing specific fungal stains [6].

Liposomal amphotericin B is the treatment of choice for disseminated and widely spread blastomycosis cases especially in immunocompromised patients who are critically ill. Itraconazole is the treatment of choice in patients with localized disease [7]. Our patient received a long course of itraconazole therapy (200 mg orally twice daily) due to the extensive involvement of his spine.

This case is unique in several respects. First, it is an unusual disease in our region and was at first thought to be a cutaneous abscess but was rather a sinus tract of the chronic deep purulent collections involving the pleura and paraspinal regions. Second, there was extensive involvement of the spine including the formation of psoas abscesses and empyema. Third, this is the second case of blastomycosis reported in a traveler returning to Saudi Arabia.

## Conclusion

Chronic *blastomycosis* can have clinical and radiological characteristics that are similar to thoracic tuberculosis or malignancies. Early diagnosis requires a high level of suspicion because there is no distinct clinical symptom for blastomycosis. This case is an unusual presentation of *blastomycosis* with chronic multifocal suppurative infection and is the second case of *blastomycosis* reported in Saudi Arabia.

## Data Availability

All data generated or analyzed during this study are included in this article.

## Abbreviations

Spp: Species
CT: Computed tomography
I&D: Incision and drainage
Fig: Figure
WBC: White blood count
MCV: Mean corpuscular volume
HIV: Human immunodeficiency virus
PPD: Purified protein derivative test
MRI: Magnetic resonance image
T8: Thoracic vertebrae number 8
L1: Lumbar vertebrae number 1
PCR: Polymerase chain reaction
TB: Tuberculosis
